# Breakdown of Immune Tolerance in Systemic Lupus Erythematosus by Dendritic Cells

**DOI:** 10.1155/2016/6269157

**Published:** 2016-02-29

**Authors:** Xiaofeng Liao, Alec M. Reihl, Xin M. Luo

**Affiliations:** Department of Biomedical Sciences and Pathobiology, Virginia-Maryland College of Veterinary Medicine, Virginia Polytechnic Institute and State University, Blacksburg, VA 24061, USA

## Abstract

Dendritic cells (DC) play an important role in the pathogenesis of systemic lupus erythematosus (SLE), an autoimmune disease with multiple tissue manifestations. In this review, we summarize recent studies on the roles of conventional DC and plasmacytoid DC in the development of both murine lupus and human SLE. In the past decade, studies using selective DC depletions have demonstrated critical roles of DC in lupus progression. Comprehensive* in vitro* and* in vivo* studies suggest activation of DC by self-antigens in lupus pathogenesis, followed by breakdown of immune tolerance to self. Potential treatment strategies targeting DC have been developed. However, many questions remain regarding the mechanisms by which DC modulate lupus pathogenesis that require further investigations.

## 1. Introduction

Systemic lupus erythematosus (SLE) is an autoimmune disease that causes damage of multiple organs [1]. Disease activity and stages can be generally divided into three patterns—the remitting relapsing pattern, chronically active disease, and long quiescence—based on various clinical manifestations that include, but are not limited to, skin rash, arthritis, nephritis, hematological disorders, and neurological disorders [2]. During SLE pathogenesis, autoreactive T cells are activated which in turn activate autoreactive B cells to produce high affinity autoantibodies against self-antigens [3]. Immune complexes (ICs) formed by aggregation of autoantibodies and self-antigens circulate in the blood and eventually deposit in peripheral tissues where the complement system is activated, ultimately inducing the release of signals that further recruit and activate autoreactive cells to feed forward a vicious cycle of chronic inflammation. Different innate and adaptive immune cell populations, including monocytes/macrophages, neutrophils, dendritic cells (DC), and lymphocytes, are recruited into peripheral tissues following the inflammatory signals to amplify inflammation and cause tissue damage [1, 4–6].

DC were discovered as the professional antigen-presenting cells (APC) with a primary function of priming naïve T cell activation [7]. Since their discovery, our understanding of how DC contribute to immune responses has much expanded, and DC have been divided into many subpopulations with distinct phenotypes and functions [8]. Two main subpopulations are classical DC (cDC) and plasmacytoid DC (pDC). DC are developed from a series of dedicated DC progenitors [8]. Common dendritic cell progenitors (CDP) originated from macrophage dendritic cell progenitors (MDP) are the first dedicated DC progenitors that can differentiate into pre-cDC and pDC in bone marrow. Pre-cDC then migrate into lymphoid and nonlymphoid tissues to differentiate into cDC. Monocytes originated from MDP can also differentiate into cDC in lymphoid and nonlymphoid tissues [8]. Murine cDC is characterized by high expression of CD11c and MHC-II on surface, while human cDC also express nonoverlapping makers CD1c (blood dendritic cell antigen 1 or BDCA1) or CD141 (BDCA3) on different subsets besides CD11c and MHC-II. Different from cDC, murine pDCs express low level CD11c and MHC-II but are positive for B220 and Siglec-H on surface, and human pDCs are defined by the expression of MHC-II, BDCA2, and BDCA4.

Functionally, cDC are professional APC that prime naïve T cells upon antigen uptake and maturation induced by appropriate maturation signals (e.g., upon TLR ligation). Mature cDC start to prime naïve T cells with the interaction between MHC-II-peptide complex on cDC and T cell receptor on T cells. The ligation of costimulatory molecules, with CD80 and CD86 on cDC and CD28 on T cells, further mutually activates cDC and T cells. Finally the cytokines secreted by cDC induce the differentiation of naïve T cells into different effector helper T cell subsets. pDCs, on the other hand, are professional interferon *α* (IFN*α*) producing cells that, through producing a high level of IFN*α*, activate multiple immune cell populations that express type I IFN receptor (IFNAR) [9]. Interestingly, pDC can also upregulate MHC-II upon activation and act like cDC to activate T cells [10].

Both cDC and pDC are important for immune tolerance to self [8]. Immature cDC when presenting self-antigens in the absence of maturation stimuli express low level MHC-II on the surface and induce immune tolerance to self. Upon activation by maturation stimuli, however, cDC mature with upregulation of MHC-II and activation markers (CD40, CD80, CD86, PD-L1, PD-L2, etc.) to facilitate inflammation. For pDCs, while their primary function is to control infections, pDCs in thymus are involved in the negative selection to maintain the central tolerance. Not surprisingly, studies have shown that both cDC and pDC play important roles in the development of autoimmune diseases, such as SLE [11].

Peripheral blood mononuclear cells (PBMC) from SLE patients can be used to study* in vitro* DC responses. Whilst important, information obtained from blood cells is limited. To this end, lupus-prone mouse models that develop lupus-like symptoms spontaneously or artificially can be used to better understand DC-mediated mechanisms of lupus progression under both* in vivo* and* in vitro* conditions. In this review, we summarize recent results obtained from studies of SLE patients and lupus-prone mice on the roles of cDC and pDC in lupus development.

## 2. *In Vivo* DC Depletion Studies: Indication of DC Involvement in Lupus 

A direct strategy to study whether a cell population is critical for the development of a disease is to deplete the population* in vivo*. Depletion of DC in wild-type mice and lupus-prone mice shows differential contributions of DC to immune homeostasis, with a tolerogenic role of DC in wild-type mice versus an immunogenic role of DC in lupus-prone mice. In wild-type mice, constitutive depletion of CD11c^high^ cDC showed normal development of regulatory T (Treg) cells and normal negative selection of CD4^+^ T cells in the thymus without an autoimmune response [12]. Constitutive depletion of both cDC and pDC in wild-type mice, however, led to increased autoimmune inflammation with elevated autoantibodies, increased IFN*γ*/IL-17-secreting T cells in peripheral tissues, and abnormal negative selection of CD4^+^ T cells in the thymus [13]. This suggests that pDC, or the combination of pDC and cDC, may contribute to immune tolerance to self. Interestingly, regardless of the presence of pDC, the absence of cDC consistently resulted in dramatic expansion of myeloid cells, particularly neutrophils and macrophages [12, 13].

In MRL/lpr lupus-prone mice, constitutive depletion of cDC and pDC did not influence the negative selection of T cells in the thymus. However, it led to fewer splenic Treg cells and less CD25 expression on the surface of these cells, suggesting compromised immune tolerance in MRL/lpr mice in the absence of DC [14]. Importantly, even though myeloid cells expanded dramatically as in wild-type mice [12, 13], glomerulonephritis and dermatitis were significantly reduced with DC depletion in MRL/lpr mice, which was accompanied by a significant decrease of the proliferation of total T cells and IFN*γ*-producing effector T cells. The lack of DC also led to significantly fewer plasmablasts and impaired autoantibody production and class switching to IgG, the primary autoantibody isotype in lupus [14]. These results demonstrate a critical role of DC in promoting lupus-like disease in MRL/lpr mice. Interestingly, the initiation of T cell activation in lupus may be DC-independent, as the ratio of naïve to activated T cells in the spleen did not change with DC depletion. It appears that autoreactive B cells, instead of DC, initiate the activation of autoreactive T cells through antigen presentation in MRL/lpr mice [15]. These data suggest that although DC can maintain immune tolerance to self in wild-type mice, overall their functions have switched to promoting autoimmune responses in lupus-prone mice.

For pDC, early transient depletion of these cells from BXSB (Yaa) lupus-prone mouse model inhibited type I IFN signature, reduced T and B cell activation, decreased autoantibody production, and improved lupus nephritis [16]. While pDC reappeared later on, the effect of early depletion was sustained, suggesting that pDCs contribute to lupus disease at the initiation stage. This observation has been confirmed by another study using B6.Nba2 lupus-prone mice [17].

These depletion studies indicate the importance of cDC and pDC in the development of lupus. Therefore, we will next summarize in detail how cDC and pDC, respectively, break down immune tolerance to self and facilitate lupus progression.

## 3. Breakdown of Immune Tolerance to Self in SLE by cDC

### 3.1. Changes of cDC Number and Phenotype in Lupus

Changes of cell number and phenotype may reflect changes of the cells' activation status and/or their dynamic trafficking into different tissues. Studies on the changes of cDC number and phenotype in lupus will help us understand whether cDC are activated and where they function to break down self immune tolerance. In SLE patients, a general sense is that cDC number and frequency in the blood are lower with higher disease activity [18–23]. The decrease of blood cDC may be due to increased migration of cDC into peripheral tissues. For example, more cDC were found to infiltrate the tubulointerstitial region in the kidney biopsy of SLE patients with proliferative or active nephritis than the healthy control (HC) or patients with nonproliferative nephritis, and the increase in renal infiltration was accompanied by a decrease of cDC number in the peripheral blood [20, 24]. Murine cDC, particularly those expressing CD11b, also accumulated in the kidney of various types of lupus-prone mice as lupus nephritis progressed [25–27]. In addition, we and others showed increased cDC accumulation in the spleen and lymph nodes of lupus-prone mice [28–32]. How cDC infiltrated inflamed tissues is unclear, but studies have shown that chemokine receptors chemR23 and CCR7 may be important for cDC migration into the kidney and secondary immune tissues, respectively [32–35]. Renal expression of chemerin—the chemokine ligand of chemR23—and increased chemR23^+^ DC in the kidney of SLE patients suggest chemerin-dependent migration of cDC into inflamed kidney in lupus [34]. CCR7, on the other hand, mediates migration of cDC to lymph nodes. Upon IFN*α* priming and lipopolysaccharide (LPS) stimulation, monocyte-derived cDC (moDC) from SLE patients expressed a significantly higher level of CCR7 [35]. Besides IFN*α* and LPS, ICs can also induce the migration of moDC towards CCR7 ligands both* in vitro* and* in vivo* [32].

The phenotype of cDC is different between tolerogenic cDC, which suppress inflammation, and immunogenic cDC that stimulate inflammation. cDC in the blood of SLE patients or secondary immune tissues of lupus-prone mice have been shown to exhibit elevated expression of CD40, CD80, CD86, PD-L1, and PD-L2, suggesting that cDC in lupus may be activated and immunogenic [18, 36–38]. However,* in vitro* studies using moDC from SLE patients or lupus-prone mice have shown inconsistent results regarding the activation phenotype of cDC [18, 36, 39–41]. Some showed higher activation state of moDC and enhanced T cell activation with lupus, while others showed either comparable activities or reduced moDC and T cell activation. The inconsistency may be due to different methods used for moDC differentiation, maturation, and activation, as different amounts of granulocyte macrophage colony-stimulating factor (GM-CSF) and IL-4 were used to generate immature moDC, and different stimuli (e.g., LPS, TNF*α*, CpG, or IFN*α*) were used to mature or activate moDC in different studies.

### 3.2. MoDC in Lupus

Monocytes can differentiate into cDC under both steady state and inflammatory state* in vivo* [8]. GM-CSF and IL-4 can also induce moDC* in vitro* [42]. However, whether monocytes are a precursor of cDC in lupus is still an open question. Monocytes incubated with sera from SLE patients could differentiate into cDC, but the differentiation depended on the presence of IFN*α* in the serum [43]. Later studies also showed that IgG-containing ICs in the serum, tumor necrosis factor (TNF) receptor I on monocytes, and the interaction between monocytes and T cells are all important for the differentiation of monocytes into cDC in lupus [35, 43, 44]. Regarding the function of differentiated moDC, only those generated in the presence of SLE sera, rather than moDC generated by IFN*α*/GM-CSF alone, could promote differentiation of IgG- and IgA-producing plasmablasts from B cells. This suggests that factors other than IFN*α* in the SLE patient sera affect the function of moDC in lupus [45].

### 3.3. Regulation of cDC Activation in Lupus

As discussed earlier, activated cDC accumulate in lymphoid and nonlymphoid tissues during lupus progression. It is important to understand how they are activated in the context of lupus.* In vitro* studies suggest that self-DNA and/or self-RNA containing antigens could activate cDC [46–48].* In vitro* generated moDC from both healthy human PBMC and wild-type mouse bone marrow can be activated by necrotic or apoptotic cell particles containing self-DNA and self-RNA to produce inflammatory cytokines (IL-6, TNF*α*), upregulate MHC-II and costimulatory molecules (CD40, CD80, and CD86), and activate allogeneic T cells that in turn produce IL-2, IFN*γ*, and IL-17. It has been demonstrated that cDC generated* in vitro* or isolated directly from human or mouse could be activated by DNA- and RNA-containing self-antigens through the signaling of toll-like receptor (TLR)9 and TLR7/8, respectively [49–54]. However, it is still unclear whether cDC can be activated by nucleic acid-containing self-antigens* in vivo*, because natural IgM antibodies and complement C1q-opsonized apoptotic particles, both present* in vivo* but not necessarily in* in vitro* experiments, have the ability to suppress cDC activation [55–57]. The suppression of p38 MAPK phosphorylation by MAPK phosphatase-1 appears to be important for cDC tolerance induced by natural IgM [56].

Studies using gene knockouts in mice have shown that TLR7, MyD88, and interferon regulatory factor (IRF)5 are important for cDC activation in lupus, and TLR8, A20, Lyn, B lymphocyte-induced maturation protein-1 (Blimp1), and Bim can downregulate cDC activation [58–65]. While TLR7 promotes cDC activation in lupus, TLR8 downregulates TLR7 expression and TLR7-dependent cDC activation [58]. IRF5-deficient cDC exhibited a reduced ability to produce TNF*α*, IL-6, and IL-10 in lupus-prone mice [61]. DC-specific deficiency of A20, Lyn, or Blimp1 led to lupus-like disease in mice [60, 62–64]. cDC isolated from Bim^−/−^ mice compared to wild-type mice induced higher T cell proliferation* in vitro*, and autoantibodies can be generated in non-lupus-prone mice upon transfer of Bim-deficient cDC [65]. The role of MyD88 in lupus cDC is debated. One study using MyD88-deficient MRL/lpr mice showed no obvious change of lupus nephritis [59], while another study using DC-specific MyD88 and Lyn double-deficient mice showed attenuated lupus disease compared to DC-specific Lyn-deficient mice [60]. Interestingly, polymorphisms within* TLR7*,* IRF5*,* TLR8*,* A20*,* Lyn*, and* Blimp1* gene loci have all been shown to be associated with SLE [66–70].

Activation of cDC can be regulated by several additional factors according to studies of SLE patient samples. Expression of immunoglobulin-like transcript (ILT)3, an inhibitory receptor, was found to be decreased on circulating cDC of SLE patients, and the decrease was correlated with higher levels of proinflammatory cytokines (type I IFN, TNF*α*) in the plasma of these patients [71]. Not surprisingly, SLE-susceptible single nucleotide polymorphisms were identified in the* ILT3* gene locus. Sex hormones may also affect the activation of cDC. In a minichromosome maintenance protein (MCM)6 dependent manner, 17beta-estradiol, a female hormone, could induce upregulation of CD40 on* in vitro*-generated moDC that in turn increased T cell activation [72]. cDC purified from SLE patients compared to HC expressed a higher level of MCM6, and MCM6 expression was positively correlated with the level of 17beta-estradiol in the sera of SLE patients [72]. Moreover, cDC activation is affected by complement C1q, although the effect of complement C1q on cDC is still unclear. One study showed that immobilized C1q coated on plates induced maturation of immature moDC differentiated* in vitro* from healthy PBMC by GM-CSF/IL-4 [73]. Mature moDC, compared to immature moDC, had increased production of IL-12, TNF*α*, and IL10 and enhanced T cell proliferation and secretion of IFN*γ*. However, another study showed that, when immobilized C1q was added concurrently with GM-CSF/IL-4 during moDC differentiation from PBMC, moDC stayed at immature state [74]. Upon LPS or LPS/IFN*γ* stimulation, these moDC did mature, but they produced less IL-12, TNF*α*, and IL-6 but more IL-10 [74]. Mature cDC generated by LPS or LPS/IFN*γ* also had reduced ability to activate T cells. The timing of C1q addition appears to be important, and further studies are required to uncover the roles of C1q in regulating cDC maturation and activation.

Apoptosis of activated cDC is important for immune tolerance to self. Under normal condition, activated cDC undergo apoptosis through either Fas-dependent or mitochondria-dependent pathways, the latter by interacting with activated Treg cells that express lymphocyte-activation gene (LAG)3 [75, 76]. DC-specific deficiency in either Fas-dependent or Fas-independent apoptosis in mice could induce lupus-like symptoms, suggesting that abnormal accumulation of activated cDC may contribute to breakdown of self-tolerance and lupus development [75–77].

### 3.4. Activation of T Cells and B Cells by cDC in Lupus

Upon activation by self-antigens, cDC can promote lupus development by interacting with T cells and B cells. While* in vivo* studies of how cDC affect autoreactive T cells are still lacking,* in vitro* evidence suggests that moDC derived from the bone marrow of lupus mice or from PBMC of SLE patients, upon activation, can promote T cell activation and hamper Treg response [39, 52, 78–80]. It is demonstrated in both mouse and human cell studies that moDC activated by apoptotic cells or cytosolic dsDNA could induce the activation of T cells, including that of autoreactive T cells [52, 79]. In addition, compared to bone marrow-derived macrophages, bone marrow-derived cDC (BMDC) from lupus-prone mice possessed higher ability to activate autoreactive T cells, suggesting that cDC rather than macrophages are the APC for autoreactive T cell activation [78]. Moreover,* in vitro* generated tolerogenic BMDC from SLE patients were less capable of generating Treg cells* in vitro* than HC BMDC [80]. Furthermore, LPS-activated BMDC from lupus-prone mice suppressed Treg function by producing more IL-6, which indirectly promoted proliferation of CD4^+^ T cells [39].

Several studies using* in vitro* systems have indicated possible roles of cDC in promoting autoreactive B cell activation [45, 52, 81–83]. A couple of them have shown that GM-CSF/IL-4-induced BMDC from B6.Sle1.Sle2.Sle3 lupus-prone mice, compared to BMDC from B6 mice, promoted better B cells proliferation and IgM/IgG production in* in vitro* coculture system upon anti-CD40 ligation [81, 82]. The enhancement was partially dependent on elevated IL-6 and IFN*γ* produced by activated BMDC. In addition, upon i.p. injection of ICs, splenic CD11c^+^ DC from B6.Sle1.Sle2.Sle3 mice produced more IL-6 and IFN*γ* than those from B6 mice. In human cell studies, moDC derived from healthy PBMC* in vitro* activated by either the sera from SLE patients or cytosolic dsDNA promoted B cell antibody class switch to IgG and IgA [45, 52]. Contradictory to these observations, however, one study showed that BMDC from several lupus-prone mouse models, when activated by LPS, possessed reduced IL-6-producing ability compared to BMDC from B6 mice [83]. Due to the decrease of IL-6 production, LPS-activated BMDC from MRL/lpr mice failed to suppress autoreactive IgM production by B cells. The discrepancy may have been due to different lupus-prone mouse models used or different activation methods (anti-CD40 versus LPS), although another study has shown that LPS could increase IL-6 production from BMDC of B6.Sle1.Sle2.Sle3 mice [39].

Besides activating T cells and B cells, cDC may also promote lupus development by producing high-mobility group box 1 (HMGB1) protein that not only binds nucleosomes to facilitate activation of cDC as a positive feedback but also enhances IFN*α* production by pDC, the latter of which will be discussed below [46, 49, 84].

### 3.5. Potential Treatment Strategies of Lupus by Targeting cDC

Since cDC can promote lupus development, they are a potential target for the development of new drugs against lupus. To target innate immune cells such as cDC, nanogel-based immunosuppressive drugs have been tested in lupus-prone mice that led to prolonged survival and reduced lupus nephritis [85, 86]. The lipid coating of nanogel enables better uptake of the drug by cDC, thus increasing the amount of immunosuppressive drug inside the cells. In addition,* in vitro* studies have shown that BMDC incubated with immunosuppressive drug-containing nanogel had lower production of inflammatory cytokines compared to cells incubated with free drug. The ability of pDC to produce IFN*α* was also suppressed, with less IFN*α* produced in the presence of nanogel [85]. It appears that cDC-targeted therapies may benefit from nanogel-based delivery with minimal side effects.

Efforts have been made to induce the generation of tolerogenic cDC to ameliorate lupus. Several studies have shown that tolerogenic cDC generated by transgenic method or induced* in vitro* can rebuild immune tolerance to self after adoptive transfer to lupus-prone mice [87–89]. Tolerogenic cDC can also be induced from PBMC of SLE patients* in vitro* to suppress T cell activation [18, 90].

## 4. Breakdown of Immune Tolerance to Self in SLE by pDC

### 4.1. Changes of pDC Number and Phenotype in Lupus

pDCs play an important role in lupus development in addition to cDC. Human studies of pDC frequency and number in the blood of SLE patients have shown inconsistent results [19–21, 91–95]. The inconsistency may reflect the dynamic change of cell number and migration of pDC corresponding to different disease stages and/or treatments. The decrease of pDC in the circulation of some SLE patients may indicate increased migration of the cells into peripheral tissues. Notably, increased infiltration of pDC to the kidney of SLE patients has been confirmed by several studies [20, 24, 95], although the location of the infiltrate is still a matter of debate. It has been suggested that pDC may use IL-18 receptor and chemR23 to migrate into the inflamed kidney that expresses IL-18 and chemerin, respectively [33, 34, 95]. In mice, however, one study showed no change of pDC in the kidney as lupus progressed [27]. pDC can also accumulate in the skin of SLE patients and lupus-prone mice [96, 97]. In MRL/lpr lupus-prone mice, UVB irradiation induces skin infiltration of pDC, while IFN*α* response in the skin has been shown to be positively correlated with the level of chemerin that can attract pDC through chemR23 [97].

Conversely, the increase of pDC in the circulation of some SLE patients may be due to increased generation and emigration of pDC from the bone marrow. Our study using MRL/lpr mice demonstrated that the number of pDC was increased in the bone marrow compared to MRL control mice [28]. A higher percentage of pDC was also found in the bone marrow of SLE patients compared to HC [98]. It is worth noting that phenotypic identification of pDC varies from one study to another and that the surface markers used to define pDC in healthy individuals may not be appropriate under the disease environment [99].

However, we and others have consistently observed the expansion of pDC in secondary immune tissues during lupus progression. We have found that pDC are increased in the MLN of young MRL/lpr mice compared to age-matched MRL controls [28]. Others using NZB/W F1 mice and NZM2328 mice have found similar results in MLN and renal lymph nodes [38, 100]. pDCs also accumulate in the spleen of lupus-prone mice, particularly in the marginal zone (MZ) of the spleen [30, 38, 82, 101, 102]. The increase of pDC in secondary lymph tissues on one hand may be caused by inflammation-induced migration and/or self-expansion* in situ*, as will be discussed later. On the other hand, pDCs appear to be able to survive better in lupus [102–104], as their expression of antiapoptotic Bcl-2 was found to be increased [102]. Survival signal in pDC from both humans and lupus-prone mice is activated by TLR7/9-induced NF*κ*B pathway [103, 105]. pDCs in lupus are constantly stimulated by TLR7/9 ligands, which are known to suppress miR-29b and miR-29c, allowing for upregulation of the target of these microRNAs, including Bcl-2 [104].

Many functional markers expressed on pDC are altered in SLE patients and lupus-prone mice. The expression of three inhibitory receptors, BDCA2, leukocyte-associated immunoglobulin-like receptor 1 (LAIR-1), and ILT3, on human pDC is reduced in SLE patients compared to HC [94, 106, 107]. On the contrary, MHC-II and costimulatory molecules are increased on pDC of both SLE patients and lupus-prone mice, suggesting an increased ability to present self-antigens and activate autoreactive T cells [28, 37, 38, 101, 108, 109].

### 4.2. Critical Roles of IFN*α* in Lupus Development

One major function of pDC in immune responses against foreign pathogens is to produce a large amount of type I IFN. Many studies have shown that type I IFN, particularly IFN*α*, is critical for lupus development. It is well known that SLE patients have elevated serum IFN*α* level that is positively correlated with disease severity [43]. Administration of IFN*α* into humans for antivirus or antitumor treatment, or into preautoimmune lupus-prone mice, can induce or accelerate lupus-like symptoms [110–112]. Deficiency of the receptor of type I IFN and IFNAR in several lupus-prone mouse models resulted in ameliorated lupus symptoms [100, 113, 114]. Interestingly, anti-IFNAR treatment transiently ameliorated lupus disease in MRL/lpr mice, but constitutive depletion of IFNAR in the same model deteriorated lupus symptoms [115, 116]. IFN*β* deficiency in BXSB mice failed to modify lupus progression, indicating that the IFN*α* subtype is the principal type I IFN important for lupus development [116]. Recent studies have shown that by either depleting pDC or abrogating IFN*α* production of pDC, lupus disease is reduced [16, 17, 117]. However, only the depletion of pDC or blockade of IFN*α* signaling at early stage of disease could prevent lupus development [17, 116]. Together, these studies suggest that through secreting IFN*α*, pDC may play a critical role in the development of lupus disease at the early initiation stage.

Many types of leukocytes can express IFNAR on the surface and respond to IFN*α*, including monocytes, cDC, pDC, T cells, and B cells [116]. Sera from SLE patients can induce normal monocytes to differentiate into cDC in an IFN*α*-dependent manner [43]. Differentiated cDC can subsequently activate both allogeneic and autologous CD4^+^ T cells. IFN*α* can also expand splenic cDC, particularly CD11b^+^CX3CR1^+^ cDC, that may have been derived from monocytes [30]. In addition, IFN*α* is able to precondition the immunogenic status of monocytes. Without IFN*α* priming, monocytes incubated with RNA-containing ICs from SLE patients failed to upregulate activation markers [118]. The same phenomenon was observed for moDC differentiated by apoptotic blebs or apoptotic cells, where IFN*α* priming enabled these moDC, which were tolerogenic without IFN*α*, to activate T cells [119, 120]. The molecular mechanism of how IFN*α* activates monocytes is still unclear, but studies have shown increased expression of two IFN*α* inducible genes, Ifi202 in bone marrow-derived DC from lupus-prone mice and Ifit4 in monocytes from SLE patients [121, 122]. Overexpression of these genes can activate normal moDC with enhanced IL-12 production, which promotes Th1 differentiation. Besides activation, IFN*α* also affects the migration of moDC. IFN*α*/GM-CSF-induced rather than IL-4/GM-CSF-induced moDC from healthy human PBMC can upregulate MMP-9 and migrate towards CCL5 and CCL3 that are expressed in inflamed tissues [123].

IFN*α* also influences pDC themselves as well as non-monocyte-derived cDC. In lupus-prone mice, IFN*α*-dependent expansion of splenic pDC has been documented [30]. With IFNAR-I deficiency, both cell number and surface activation markers of splenic pDC were reduced [100]. In the case for non-monocyte-derived cDC, studies of IFNAR-I-deficient NZM2328 mice have shown reduced splenic CD8^+^ and CD8^−^ cDC with decreased activation markers [100]. IL-12- and TNF*α*-producing ability of CD8^+^ cDC was also reduced in the absence of IFNAR-I [100].

Regarding T cells, an* in vitro* study showed that normal cDC primed by IFN*α* could promote naïve T cells to differentiate into Th1/Th17 T cells [124]. However, if IFN*α* was constantly present in the cDC-T cell coculture system, it had a suppressive effect for Th1/Th17 differentiation. IFN*α* can also promote inflammatory T cell function by inducing the migration of effector T cells into inflamed tissues in a CXCR3-dependent manner [125].

Studies on lupus-prone mouse models have shown that IFN*α*-producing pDC can directly influence autoreactive B cell response. In BXD2 lupus-prone mice, it was demonstrated that the accumulation of activated pDC in the MZ of spleen resulted in the upregulation of CD86 on MZ B cells, which was important for germinal center (GC) formation and autoantibody production [126]. In addition, MZ B cells increased their migration into the follicular region in response to IFN*α* produced by the accumulated pDC. Such migration of B cells reduced the interaction with MZ macrophages, causing the macrophages to decrease in number in the MZ [127]. This would compromise clearance of apoptotic cells in the spleen of lupus-prone mice and promote exposure of autoantigens to DC, autoreactive T cells, and B cells.

### 4.3. Regulation of IFN*α* Production from pDC in Lupus

Due to the critical role of IFN*α* in lupus development, how pDC are activated to produce IFN*α* in lupus has been studied. pDCs produce a large amount of IFN*α* upon TLR7 and TLR9 stimulation by bacterial or viral nucleic acids [8]. Thus, infections could be a trigger of IFN*α* production by pDC in lupus. One study showed that Epstein-Barr virus (EBV) infection was associated with lupus [128]. In addition, nucleic acid self-antigens and/or nucleic acid-containing ICs are another potential inducer of TLR7/9-dependent IFN*α* production by pDC in lupus [128]. Nucleic acid self-antigens derived from apoptotic or necrotic cells are increased significantly in SLE patients and lupus-prone mice compared to respective controls [1]. When the sera of SLE patients were mixed with healthy PBMC, more IFN*α* production was induced from pDC [129]. The patient sera contained ICs formed between IgG and apoptotic cells, which were found to activate pDC to produce IFN*α* through TLR7/9 [53, 130–133]. Interestingly, IgG alone or ICs with nucleic acid digestion failed to induce IFN*α* production by normal pDC, suggesting a critical role of TLR7/9 stimulation by nucleic acids within the ICs. However, DNA/RNA alone or nucleic acid-containing ICs in the presence of Fc*γ*RAII blockade also could not trigger pDC to produce IFN*α*, indicating that the interaction between IgG in ICs and Fc*γ*RAII on pDC is important for IC-induced IFN*α* production by pDC [130, 133]. Moreover, it has been shown that CpG motif in dsDNA of DNA-containing ICs is required for IFN*α* production by normal pDC [50].

Nucleic acid self-antigens can also induce IFN*α* production by pDC in an Fc receptor- (FcR-) independent pathway free from the formation of ICs. LL37, an antimicrobial peptide, has been shown to complex with self-DNA and self-RNA to form nanoscale aggregates that trigger IFN*α* production by normal pDC in a TLR7/9-dependent manner [54, 134]. Neutrophils from SLE patients possess an increased ability to release neutrophil extracellular traps (NETs), which contain LL37 [108, 135]. When LL37 was digested, NETs were no longer able to induce IFN*α* production by pDC, suggesting a critical role for this peptide [135]. IFN*α* in turn can upregulate LL37 and HNP (another antimicrobial peptide) on the surface neutrophils as seen in the blood of SLE pateints [108]. The levels of anti-LL37 and anti-HNP antibodies in the patient sera are also increased, which, when ligated with transmembrane expressed LL37 and HNP, respectively, can trigger the release of NETs by neutrophils. These results suggest that a positive feedback loop between NETs release by neutrophils and IFN*α* production by pDC may initiate and/or promote lupus development in SLE patients. Interestingly, LL37 has been found to be also important for Fc*γ*RIIA-dependent IFN*α* production from pDC, likely through facilitating the internalization of ICs [135].

Signaling molecules in the TLR7/9 pathway are important for autoantigen-induced IFN*α* production from pDC. SLC15A4-, MyD88-, IRF8-, or IRF5-deficient lupus-prone mice have shown ameliorated lupus symptoms with reduced IFN*α* protein level in the serum, decreased IFN*α* transcript level in pDC, downregulation of type I IFN inducible genes, and suppressed activation of both T cells and B cells [59, 61, 136–138]. In addition, pDC from IRF-5- or IRF7-deficient mice failed to produce IFN*α* upon stimulation with RNA-containing ICs from the sera of SLE patients [50, 139]. Moreover, interleukin-1 receptor-associated kinase (IRAK)1 and IRAK4 are required for IFN*α* induction from pDC, as their inhibition abrogates the production of IFN*α* from healthy pDC stimulated with the sera of SLE patients [140].

The ability of pDC to produce IFN*α* is also regulated by many other factors that may influence the outcome of lupus development. High-mobility group box (HMGB) proteins, for example, function as universal sentinels for nucleic acid-mediated immune response through both cytosolic receptors and those in endosomes including TLR9 and TLR7 [141]. It has been shown that, compared to CpG-A alone, HMGB1-bound CpG-A could induce higher IFN*α* and TNF*α* production by normal pDC [142]. This is due to increased recruitment of MyD88 to TLR9 in the presence of HMGB1. In addition, HMGB1 can facilitate the formation of CpG-TLR9 complexes and retain the complexes in early endosome rather than lysosome, resulting in sustained IFN*α* production by pDC [49]. Studies on SLE patient samples have shown that the level of HMGB1 in the circulation was positively correlated with the concentration of IFN*α* [46, 107]. Moreover, the interaction between HMGB1 and receptor for advanced glycation endproducts (RAGE) is required, as PBMC from HC incubated with the sera of SLE patients produce much less IFN*α* when the interaction is blocked [46, 142].

Amyloid fibrils can also regulate IFN*α* production from pDC by modulating the trafficking of nucleic acid-TLR complexes. These are stable insoluble aggregates of misfolded protein products with extensive *β*-sheet structure that can facilitate the maintenance of nucleic acid antigens in early endosomes of pDC [143]. Albeit rare, amyloid fibrils have been found to be associated with SLE cases and complicate lupus nephritis [144]. Immunization of healthy mice with DNA-containing amyloid fibrils induces lupus-like disease, promoting autoantibody production and lupus nephritis [143].

C-reactive protein (CRP), an acute-phase reactant produced by liver in response to inflammation, can suppress IFN*α* production from normal pDC by increasing the trafficking to ICs into late endosomes in pDC [132]. Therefore, CRP may be beneficial for lupus disease through inhibiting IFN*α* production. In SLE patients, the elevation of CRP in response to inflammation is modest and much less than expected, suggesting compromised regulation of IFN*α* production [145].

Complement C1q is another suppressive factor of IFN*α* production from pDC. Human individuals with C1q-deficiency can develop SLE [146, 147]. When C1q is added simultaneously, RNA-containing ICs or CpG stimulated less production of IFN*α*, IL-6, IL-8, and TNF*α* from PBMC or purified healthy pDC [148]. The suppressive effect of C1q on IFN*α* production from pDC has been shown to be dependent on the ligation of C1q to LAIR-1 expressed on pDC [149].

Sex hormones may also regulate IFN*α* production from pDC in SLE patients. One study has shown that that TLR7 agonist induced higher IFN*α* production by PBMC from healthy women than those from healthy men [150]. In addition, 17beta-estradiol, a female hormone, can increase IFN*α* production from pDC upon CpG stimulation [151].

pDC may interact with other cell types* in vivo* that affect their ability to produce IFN*α*. Studies have shown that B cells, platelets, NK cells, and monocytes can differentially influence IFN*α* production by pDC [152–156]. B cells facilitate IFN*α* production by normal pDC stimulated with RNA-containing ICs or CpG-A [152]. Interestingly, the mechanisms of B cell involvement are different depending on the stimulation. For RNA-containing ICs, the contact between B cells and pDC through adhesion molecule CD31 is required, while the elevation of CpG-induced IFN*α* production is B cell contact-independent. The latter may be dependent on an unknown secreted molecule, as the supernatant from CpG-A-stimulated B cell culture could promote IFN*α* production from pDC. In addition, activated platelets, found to be more abundant in the blood of SLE patients, can promote IFN*α* production from normal pDC stimulated with nucleic acid-containing ICs through interaction between CD154 on platelets and CD40 on pDC [153]. In lupus-prone mice, depletion of platelets improved, while administration of activated platelets worsened, lupus disease, suggesting the involvement of platelets in lupus development. Moreover, CD56^dim^CD16^+^ NK cells can promote IFN*α* production from pDC upon stimulation with RNA-containing ICs in the coculture of pDC and NK cells through both secreted MIP-1*β* and CD11a-dependent direct contact between the two cell types [154, 155]. NK cells isolated from SLE patients, however, produced less IFN*α* than NK cells from HC, since most of them were CD56^bright^CD16^−^ rather than CD56^dim^CD16^+^ NK cells. Furthermore, CD14^+^ monocytes, contrary to B cell, platelets, and NK cells, can suppress IFN*α* production from pDC through various mechanisms. It has been shown that upon RNA-containing ICs stimulation, CD14^+^ monocytes produced TNF*α*, prostaglandin E2, and reactive oxygen species, all of which suppressed IFN*α* production from normal pDC in coculture [155]. Additionally, monocytes can suppress IFN*α* production from normal pDC through competitive binding of C1q-coated ICs to reduce internalization of ICs in pDC [156]. Monocytes isolated from SLE patients have less suppressive effect on IFN*α* production from pDC compared to those isolated from HC [155].

### 4.4. IFN*α*-Producing Ability of pDC in Lupus

While the essential role of IFN*α*-producing pDC in lupus is inarguable, questions remain on whether pDCs are the major IFN*α*-producing cells during the entire course of lupus progression. It has been demonstrated in several studies that PBMC or pDC purified from PBMC of SLE patients produced much less IFN*α* upon TLR9-ligand stimulation compared to HC [93, 157–159]. Similar results have been obtained from lupus-prone mice [101]. We have shown in our recent study that pDC isolated from older MRL/lpr mice in the late stage of lupus development produced significantly less IFN*α* upon CpG stimulation* in vitro* compared to pDC purified from younger mice in the early stage [109]. The reduced IFN*α*-producing ability may be due to continuous exposure to nucleic acid self-antigens, as pDC from HC produced much less IFN*α* after repeated stimulation with CpG or DNA-containing ICs [159]. Notably, one study showed comparable IFN*α* production between pDC from SLE patients versus healthy individuals [160]. In their study, however, IL-3 was added in cell culture medium, which may have enhanced IFN*α* production by pDC from SLE patients. Resting or the addition of IFN*α*, IFN*γ*, and GM-CSF could also recover IFN*α*-producing ability of pDC from SLE patients to some extent [157, 159]. This suggests that the deficiency of IFN*α* production from pDC is reversible. Moreover, IFN*α* production by pDC from SLE versus HC was comparable upon stimulation with influenza viruses or TLR7 agonist [43, 158]. It is possible that pDC in SLE patients and lupus-prone mice can still produce a normal level of IFN*α* through the TLR7 pathway. Collectively, the results of these studies have raised two important questions: (1) Do pDCs gradually lose the ability to produce IFN*α in vivo* during lupus progression? (2) If pDC fail to produce IFN*α* in late stage lupus, what is the source of IFN*α* that stays at a high level in SLE patients and lupus-prone mice? [94].

### 4.5. Possible IFN*α* Production from Cells Other Than pDC in Lupus

An early study showed that PBMC from SLE patients could still produce detectable IFN*α* when pDCs were depleted, suggesting that other cell types besides pDC may have the ability to produce IFN*α* in SLE [43]. Neutrophils isolated from HC, SLE patients, and B6 mice were able to do so upon nucleosomes or CpG-B stimulation [161]. Interestingly, neutrophils from TLR9-deficient mice retained their ability to produce IFN*α* upon nucleosomes stimulation, suggesting that the production IFN*α* in neutrophils is TLR9-independent. Moreover, neutrophils from both SLE patients and lupus-prone mice possessed increased IFN*α* transcript level compared to HC, although the protein level of IFN*α* was not measured in these studies [162–164]. Besides neutrophils, monocytes and cDC can also produce IFN*α*. With IFN*β* priming, monocytes purified from healthy human PBMC, as well as cDC derived* in vitro* from bone marrow of normal mice, were shown to produce IFN*α* through LPS-activated TLR4 pathway [165]. Monocytes from healthy human PBMC also produced IFN*α* upon stimulation with liposome-coated RNA [166]. In addition, Ly6C^high^ monocytes are the primary source of IFN*α* in pristine-induced lupus-prone mice, as depletion of these monocytes abrogated IFN*α* production [167]. cDC, on the other hand, have been shown to produce IFN*α* through a cytosolic pattern recognition pathway via stimulator of interferon genes (STING) [168].

### 4.6. Potential Treatment Strategies of Lupus by Targeting pDC and IFN*α*

Due to the critical role of pDC and IFN*α* in the development of lupus, potential treatment strategies targeting them have been proposed. One example is intravenous immunoglobulin (IVIG) therapy, where IgG, the major antibody in IVIG, inhibits IC- or CpG-A-mediated production of IFN*α* from pDC [129]. It has been suggested that Fc fragment of IgG through blocking Fc*γ*RIIA on pDCs directly suppresses the uptake of nucleic acid-containing ICs by pDC [169]. Through the function of IgG glycan hydrolysis, Endoglycosidase S (Endo S) can also inhibit the uptake of ICs [170]. Sialylated subfraction positive (SNA^+^) Fab′ fragment of IgG, targeting unknown receptor on monocytes, induces production of PGE2 by monocytes, which in turn suppresses TLR7/9 agonist-mediated IFN*α* production by pDC. Another potential treatment targeting pDC and IFN*α* is DNA-like class R inhibitory oligonucleotides (INH-ODNs), which block TRL7/9-mediated activation of pDC upon stimulation with nucleic acid-containing ICs [171]. Administration of INH-ODN in MRL/lpr lupus-prone mice dramatically ameliorated lupus disease with reduced pathology and autoantibodies. Moreover, proteasome inhibitors have been shown to suppress IFN*α* production from normal pDC by inhibiting TLR9 translocation from endoplasmic reticulum to endosomes and lysosomes [172, 173]. Furthermore, HMG-CoA reductase inhibitors (statins) and histone deacetylases inhibitors can suppress IFN*α* production by healthy human pDC through inhibiting IRF7 translocation into the nucleus [174, 175]. Lastly, by neutralizing IFN*α* directly, sifalimumab, a monoclonal antibody against human IFN*α*, was able to reduce IFN-signature in phase I clinical trial [176].

A strategy to induce tolerogenic pDC has also been proposed. Subcutaneous injection of H471-94 peptide from histone proteins into NSF1 lupus-prone mice at a low dose induced tolerogenic pDC that promoted Treg cells [177]. Adoptive transfer of tolerogenic pDC into lupus-prone mice was able to reduce autoantibodies against DNA-containing antigens, decrease IL-17 production in spleen, and delay the development of lupus nephritis [177].

## 5. Open Questions

Many questions remain regarding the mechanisms by which DC modulate lupus pathogenesis that needs to be revealed by additional studies. The first question is whether and how selective depletion of cDC would affect lupus. Many different lupus-prone mouse models have been generated, making it feasible to investigate whether DC are important for lupus development* in vivo*. Depletion studies of whole DC populations, including both cDC and pDC, in MRL/lpr lupus-prone mice suggest the involvement of DC in promoting lupus development, but not activation of naïve T cells. Two additional studies that selectively deplete pDC or abrogate IFN*α*-producing ability of pDC in lupus-prone mouse models other than MRL/lpr further demonstrate the importance of pDC in lupus pathogenesis. However, selective depletion of cDC populations in lupus-prone mice has not been reported.

The second question is which TLR, TLR9, or TLR7 is critical for the role of pDC in lupus pathogenesis. Studies have shown that the pathogenic role of TLR7 in lupus-prone mice is partially dependent on IFN*α* induction, and TLR9 on the contrary can regulate lupus progression by suppressing TLR7 signaling [178–181]. However, pDC-specific TLR7 or TLR9 deficiency in lupus-prone mice has not been reported, as B cells and some other innate immune cell types also express TLR7 and TLR9.

A third question is how to develop new treatment strategies targeting DC populations for lupus. Current treatments for lupus are nonspecific immunosuppressive drugs that suppress general immune responses from both innate and adaptive immune system. Side effects, including increases susceptibility to cancers and/or infections, can be severe. Future direction for lupus treatment should be focused on specific targeting with minimal side effects, where DC are a valuable target. New drugs targeting DC should avoid blocking the mechanism by which they defend against pathogens or cancer cells. Therefore, a better understanding of how DC are activated in lupus versus cancer/infection will be particularly useful.

How to translate results obtained from* in vitro* studies is another question. Through either purifying DC directly from PBMC of SLE patients or* in vitro* generating moDC, researchers have investigated activation of DC by self-antigens, activation/maturation markers on DC, cytokine production by DC, and the ability of DC to activate T cells. Similar studies have also been done with bone marrow cells or sorted splenic DC from lupus-prone mice. However, the results from different studies are not always consistent or even contradictory to each other, likely due to differences in stimulation protocols. It is also unclear whether* in vitro* stimulation methods would create the actual environment for DC in SLE patients or lupus-prone mice. In many cases,* in vitro* studies have revealed that the stimuli for DC activation have to be of certain concentrations or given at specific time points, making it difficult to translate the results.

## 6. Summary

Based on the reviewed studies above, we summarize how cDC and pDC may be involved in lupus pathogenesis. At the initiation stage of lupus, dysregulated cDC and pDC are activated by accumulated self-antigens (e.g., self-nucleic acids bound with associated molecules) and cytokines in genetically predisposed individuals and accumulate in peripheral immune and nonimmune tissues. Activated pDCs through secreting IFN*α* then provide immunogenic signals to other immune cells including cDC, monocytes, neutrophils, T cells, and B cells. These leukocytes further promote the activation of pDC and IFN*α* production. With increasing inflammation, monocytes differentiate into activated cDC, which, together with CDP-derived activated cDC, sustain and amplify primed adaptive immune responses in both immune and nonimmune tissues, thus exacerbating the disease.

## References

[1] Tsokos G. C. (2011). Systemic lupus erythematosus. *The New England Journal of Medicine*.

[2] Lam G. K. W., Petri M. (2005). Assessment of systemic lupus erythematosus. *Clinical and Experimental Rheumatology*.

[3] Shlomchik M. J., Craft J. E., Mamula M. J. (2001). From T to B and back again: positive feedback in systemic autoimmune disease. *Nature Reviews Immunology*.

[4] Liu Z., Davidson A. (2012). Taming lupus-a new understanding of pathogenesis is leading to clinical advances. *Nature Medicine*.

[5] Rahman A., Isenberg D. A. (2008). Systemic lupus erythematosus. *The New England Journal of Medicine*.

[6] Nowling T. K., Gilkeson G. S. (2011). Mechanisms of tissue injury in lupus nephritis. *Arthritis Research & Therapy*.

[7] Inaba K., Steinman R. M. (1985). Protein-specific helper T-lymphocyte formation initiated by dendritic cells. *Science*.

[8] Merad M., Sathe P., Helft J., Miller J., Mortha A. (2013). The dendritic cell lineage: ontogeny and function of dendritic cells and their subsets in the steady state and the inflamed setting. *Annual Review of Immunology*.

[9] Theofilopoulos A. N., Baccala R., Beutler B., Kono D. H. (2005). Type I interferons (*α*/*β*) in immunity and autoimmunity. *Annual Review of Immunology*.

[10] Grouard G., Rissoan M.-C., Filgueira L., Durand I., Banchereau J., Liu Y.-J. (1997). The enigmatic plasmacytoid T cells develop into dendritic cells with interleukin (IL)-3 and CD40-ligand. *Journal of Experimental Medicine*.

[11] Ganguly D., Haak S., Sisirak V., Reizis B. (2013). The role of dendritic cells in autoimmunity. *Nature Reviews Immunology*.

[12] Birnberg T., Bar-On L., Sapoznikov A. (2008). Lack of conventional dendritic cells is compatible with normal development and T cell homeostasis, but causes myeloid proliferative syndrome. *Immunity*.

[13] Ohnmacht C., Pullner A., King S. B. S. (2009). Constitutive ablation of dendritic cells breaks self-tolerance of CD4 T cells and results in spontaneous fatal autoimmunity. *The Journal of Experimental Medicine*.

[14] Teichmann L. L., Ols M. L., Kashgarian M., Reizis B., Kaplan D. H., Shlomchik M. J. (2010). Dendritic cells in lupus are not required for activation of T and B cells but promote their expansion, resulting in tissue damage. *Immunity*.

[15] Yan J., Harvey B. P., Gee R. J., Shlomchik M. J., Mamula M. J. (2006). B cells drive early T cell autoimmunity in vivo prior to dendritic cell-mediated autoantigen presentation. *The Journal of Immunology*.

[16] Rowland S. L., Riggs J. M., Gilfillan S. (2014). Early, transient depletion of plasmacytoid dendritic cells ameliorates autoimmunity in a lupus model. *The Journal of Experimental Medicine*.

[17] Davison L. M., Jørgensen T. N. (2015). Sialic acid-binding immunoglobulin-type lectin H-positive plasmacytoid dendritic cells drive spontaneous lupus-like disease development in B6.Nba2 mice. *Arthritis & Rheumatology*.

[18] Crispín J. C., Vargas-Rojas M. I., Monsiváis-Urenda A., Alcocer-Varela J. (2012). Phenotype and function of dendritic cells of patients with systemic lupus erythematosus. *Clinical Immunology*.

[19] Migita K., Miyashita T., Maeda Y. (2005). Reduced blood BDCA-2^+^ (lymphoid) and CD11c^+^ (myeloid) dendritic cells in systemic lupus erythematosus. *Clinical and Experimental Immunology*.

[20] Fiore N., Castellano G., Blasi A. (2008). Immature myeloid and plasmacytoid dendritic cells infiltrate renal tubulointerstitium in patients with lupus nephritis. *Molecular Immunology*.

[21] Jin O., Kavikondala S., Sun L. (2008). Systemic lupus erythematosus patients have increased number of circulating plasmacytoid dendritic cells, but decreased myeloid dendritic cells with deficient CD83 expression. *Lupus*.

[22] Khan S. A., Nowatzky J., Jiménez-Branda S. (2013). Active systemic lupus erythematosus is associated with decreased blood conventional dendritic cells. *Experimental and Molecular Pathology*.

[23] Henriques A., Inês L., Carvalheiro T. (2012). Functional characterization of peripheral blood dendritic cells and monocytes in systemic lupus erythematosus. *Rheumatology International*.

[24] Segerer S., Heller F., Lindenmeyer M. T. (2008). Compartment specific expression of dendritic cell markers in human glomerulonephritis. *Kidney International*.

[25] Schiffer L., Bethunaickan R., Ramanujam M. (2008). Activated renal macrophages are markers of disease onset and disease remission in lupus nephritis. *The Journal of Immunology*.

[26] Bethunaickan R., Berthier C. C., Ramanujam M. (2011). A unique hybrid renal mononuclear phagocyte activation phenotype in murine systemic lupus erythematosus nephritis. *The Journal of Immunology*.

[27] Sahu R., Bethunaickan R., Singh S., Davidson A. (2014). Structure and function of renal macrophages and dendritic cells from lupus-prone mice. *Arthritis and Rheumatology*.

[28] Liao X., Ren J., Wei C. H. (2015). Paradoxical effects of all-trans-retinoic acid on lupus-like disease in the MRL/lpr mouse model. *PLoS ONE*.

[29] Deane J. A., Pisitkun P., Barrett R. S. (2007). Control of toll-like receptor 7 expression is essential to restrict autoimmunity and dendritic cell proliferation. *Immunity*.

[30] Buechler M. B., Teal T. H., Elkon K. B., Hamerman J. A. (2013). Cutting edge: type i ifn drives emergency myelopoiesis and peripheral myeloid expansion during chronic tlr7 signaling. *The Journal of Immunology*.

[31] Kalled S. L., Cutler A. H., Burkly L. C. (2001). Apoptosis and altered dendritic cell homeostasis in lupus nephritis are limited by anti-CD154 treatment. *Journal of Immunology*.

[32] Clatworthy M. R., Aronin C. E. P., Mathews R. J., Morgan N. Y., Smith K. G. C., Germain R. N. (2014). Immune complexes stimulate CCR7-dependent dendritic cell migration to lymph nodes. *Nature Medicine*.

[33] Gonzalvo-Feo S., Del Prete A., Pruenster M. (2014). Endothelial cell-derived chemerin promotes dendritic cell transmigration. *Journal of Immunology*.

[34] De Palma G., Castellano G., Del Prete A. (2011). The possible role of ChemR23/Chemerin axis in the recruitment of dendritic cells in lupus nephritis. *Kidney International*.

[35] Rodriguez-Pla A., Patel P., Maecker H. T. (2014). IFN priming is necessary but not sufficient to turn on a migratory dendritic cell program in lupus monocytes. *Journal of Immunology*.

[36] Ding D., Mehta H., McCune W. J., Kaplan M. J. (2006). Aberrant phenotype and function of myeloid dendritic cells in systemic lupus erythematosus. *The Journal of Immunology*.

[37] Colonna L., Dinnal J.-A., Shivers D. K., Frisoni L., Caricchio R., Gallucci S. (2006). Abnormal costimulatory phenotype and function of dendritic cells before and after the onset of severe murine lupus. *Arthritis Research and Therapy*.

[38] Gleisner M. A., Reyes P., Alfaro J. (2013). Dendritic and stromal cells from the spleen of lupic mice present phenotypic and functional abnormalities. *Molecular Immunology*.

[39] Wan S., Xia C., Morel L. (2007). IL-6 produced by dendritic cells from lupus-prone mice inhibits CD4^+^CD25^+^ T cell regulatory functions. *Journal of Immunology*.

[40] Berkun Y., Verbovetski I., Ben-Ami A. (2008). Altered dendritic cells with tolerizing phenotype in patients with systemic lupus erythematosus. *European Journal of Immunology*.

[41] Carreno L. J., Pacheco R., Gutierrez M. A., Jacobelli S., Kalergis A. M. (2009). Disease activity in systemic lupus erythematosus is associated with an altered expression of low-affinity Fc*γ* receptors and costimulatory molecules on dendritic cells. *Immunology*.

[42] Sallusto F., Lanzavecchia A. (1994). Efficient presentation of soluble antigen by cultured human dendritic cells is maintained by granulocyte/macrophage colony-stimulating factor plus interleukin 4 and downregulated by tumor necrosis factor *α*. *The Journal of Experimental Medicine*.

[43] Blanco P., Palucka A. K., Gill M., Pascual V., Banchereau J. (2001). Induction of dendritic cell differentiation by IFN-*α* in systemic lupus erythematosus. *Science*.

[44] Deng G.-M., Liu L., Kyttaris V. C., Tsokos G. C. (2010). Lupus serum IgG induces skin inflammation through the TNFR1 signaling pathway. *The Journal of Immunology*.

[45] Joo H., Coquery C., Xue Y. (2012). Serum from patients with SLE instructs monocytes to promote IgG and IgA plasmablast differentiation. *The Journal of Experimental Medicine*.

[46] Urbonaviciute V., Fürnrohr B. G., Meister S. (2008). Induction of inflammatory and immune responses by HMGB1-nucleosome complexes: implications for the pathogenesis of SLE. *The Journal of Experimental Medicine*.

[47] Fransen J. H., Hilbrands L. B., Ruben J. (2009). Mouse dendritic cells matured by ingestion of apoptotic blebs induce T cells to produce interleukin-17. *Arthritis & Rheumatism*.

[48] Fransen J. H., Hilbrands L. B., Jacobs C. W., Adema G. J., Berden J. H., Van der Vlag J. (2009). Both early and late apoptotic blebs are taken up by DC and induce IL-6 production. *Autoimmunity*.

[49] Ivanov S., Dragoi A.-M., Wang X. (2007). A novel role for HMGB1 in TLR9-mediated inflammatory responses to CpG-DNA. *Blood*.

[50] Yasuda K., Richez C., Uccellini M. B. (2009). Requirement for DNA CpG content in TLR9-dependent dendritic cell activation induced by DNA-containing immune complexes. *Journal of Immunology*.

[51] Decker P., Singh-Jasuja H., Haager S., Kötter I., Rammensee H.-G. (2005). Nucleosome, the main autoantigen in systemic lupus erythematosus, induces direct dendritic cell activation via a MyD88-independent pathway: consequences on inflammation. *The Journal of Immunology*.

[52] Kis-Toth K., Szanto A., Thai T.-H., Tsokos G. C. (2011). Cytosolic DNA-activated human dendritic cells are potent activators of the adaptive immune response. *The Journal of Immunology*.

[53] Savarese E., Chae O.-W., Trowitzsch S. (2006). U1 small nuclear ribonucleoprotein immune complexes induce type I interferon in plasmacytoid dendritic cells through TLR7. *Blood*.

[54] Ganguly D., Chamilos G., Lande R. (2009). Self-RNA-antimicrobial peptide complexes activate human dendritic cells through TLR7 and TLR8. *The Journal of Experimental Medicine*.

[55] Vas J., Grönwall C., Marshak-Rothstein A., Silverman G. J. (2012). Natural antibody to apoptotic cell membranes inhibits the proinflammatory properties of lupus autoantibody immune complexes. *Arthritis and Rheumatism*.

[56] Gronwall C., Chen Y., Vas J. (2012). MAPK phosphatase-1 is required for regulatory natural autoantibody-mediated inhibition of TLR responses. *Proceedings of the National Academy of Sciences of the United States of America*.

[57] Clarke E. V., Weist B. M., Walsh C. M., Tenner A. J. (2015). Complement protein C1q bound to apoptotic cells suppresses human macrophage and dendritic cell-mediated Th17 and Th1 T cell subset proliferation. *Journal of Leukocyte Biology*.

[58] Desnues B., Macedo A. B., Roussel-Queval A. (2014). TLR8 on dendritic cells and TLR9 on B cells restrain TLR7-mediated spontaneous autoimmunity in C57BL/6 mice. *Proceedings of the National Academy of Sciences of the United States of America*.

[59] Teichmann L. L., Schenten D., Medzhitov R., Kashgarian M., Shlomchik M. J. (2013). Signals via the adaptor MyD88 in B cells and DCs make distinct and synergistic contributions to immune activation and tissue damage in lupus. *Immunity*.

[60] Lamagna C., Scapini P., van Ziffle J. A., DeFranco A. L., Lowell C. A. (2013). Hyperactivated MyD88 signaling in dendritic cells, through specific deletion of Lyn kinase, causes severe autoimmunity and inflammation. *Proceedings of the National Academy of Sciences of the United States of America*.

[61] Tada Y., Kondo S., Aoki S. (2011). Interferon regulatory factor 5 is critical for the development of lupus in MRL/lpr mice. *Arthritis and Rheumatism*.

[62] Kool M., van Loo G., Waelput W. (2011). The ubiquitin-editing protein A20 prevents dendritic cell activation, recognition of apoptotic cells, and systemic autoimmunity. *Immunity*.

[63] Kim S. J., Gregersen P. K., Diamond B. (2013). Regulation of dendritic cell activation by microRNA let-7c and BLIMP1. *The Journal of Clinical Investigation*.

[64] Kim S. J., Zou Y. R., Goldstein J., Reizis B., Diamond B. (2011). Tolerogenic function of Blimp-1 in dendritic cells. *The Journal of Experimental Medicine*.

[65] Chen M., Huang L., Wang J. (2007). Deficiency of Bim in dendritic cells contributes to overactivation of lymphocytes and autoimmunity. *Blood*.

[66] dos Santos B. P., Valverde J. V., Rohr P. (2012). TLR7/8/9 polymorphisms and their associations in systemic lupus erythematosus patients from Southern Brazil. *Lupus*.

[67] Kawasaki A., Kyogoku C., Ohashi J. (2008). Association of IRF5 polymorphisms with systemic lupus erythematosus in a Japanese population: support for a crucial role of intron 1 polymorphisms. *Arthritis & Rheumatism*.

[68] Musone S. L., Taylor K. E., Lu T. T. (2008). Multiple polymorphisms in the TNFAIP3 region are independently associated with systemic lupus erythematosus. *Nature Genetics*.

[69] Lu R., Vidal G. S., Kelly J. A. (2009). Genetic associations of LYN with systemic lupus erythematosus. *Genes and Immunity*.

[70] Gateva V., Sandling J. K., Hom G. (2009). A large-scale replication study identifies TNIP1, PRDM1, JAZF1, UHRF1BP1 and IL10 as risk loci for systemic lupus erythematosus. *Nature Genetics*.

[71] Jensen M. A., Patterson K. C., Kumar A. A., Kumabe M., Franek B. S., Niewold T. B. (2013). Functional genetic polymorphisms in ILT3 are associated with decreased surface expression on dendritic cells and increased serum cytokines in lupus patients. *Annals of the Rheumatic Diseases*.

[72] Xie H., Hua C., Sun L. (2011). 17*β*-estradiol induces CD40 expression in dendritic cells via MAPK signaling pathways in a minichromosome maintenance protein 6-dependent manner. *Arthritis & Rheumatism*.

[73] Csomor E., Bajtay Z., Sándor N. (2007). Complement protein C1q induces maturation of human dendritic cells. *Molecular Immunology*.

[74] Teh B. K., Yeo J. G., Chern L. M., Lu J. (2011). C1q regulation of dendritic cell development from monocytes with distinct cytokine production and T cell stimulation. *Molecular Immunology*.

[75] Stranges P. B., Watson J., Cooper C. J. (2007). Elimination of antigen-presenting cells and autoreactive T cells by Fas contributes to prevention of autoimmunity. *Immunity*.

[76] Chen M., Felix K., Wang J. (2011). Immune regulation through mitochondrion-dependent dendritic cell death induced by T regulatory cells. *The Journal of Immunology*.

[77] Chen M., Wang Y.-H., Wang Y. (2006). Dendritic cell apoptosis in the maintenance of immune tolerance. *Science*.

[78] Yang P., Li B., Lv P., Zhang Y., Gao X.-M. (2007). Interaction between antigen presenting cells and autoreactive T cells derived from BXSB mice with murine lupus. *Cell Research*.

[79] Tzeng T.-C., Suen J.-L., Chiang B.-L. (2006). Dendritic cells pulsed with apoptotic cells activate self-reactive T-cells of lupus mice both in vitro and in vivo. *Rheumatology*.

[80] Estrada-Capetillo L., Hernández-Castro B., Monsiváis-Urenda A. (2013). Induction of Th17 lymphocytes and treg cells by monocyte-derived dendritic cells in patients with rheumatoid arthritis and systemic lupus erythematosus. *Clinical and Developmental Immunology*.

[81] Wan S., Zhou Z., Duan B., Morel L. (2008). Direct B cell stimulation by dendritic cells in a mouse model of lupus. *Arthritis and Rheumatism*.

[82] Sang A., Zheng Y.-Y., Yin Y. (2014). Dysregulated cytokine production by dendritic cells modulates B cell responses in the NZM2410 mouse model of lupus. *PLoS ONE*.

[83] Gilbert M. M., Carnathan D. G., Cogswell P. C., Lin L., Baldwin A. S., Vilen B. J. (2007). Dendritic cells from lupus-prone mice are defective in repressing immunoglobulin secretion. *The Journal of Immunology*.

[84] Iwata Y., Furuichi K., Sakai N. (2009). Dendritic cells contribute to autoimmune kidney injury in MRL-*Fas*
^lpr^ mice. *The Journal of Rheumatology*.

[85] Look M., Stern E., Wang Q. A. (2013). Nanogel-based delivery of mycophenolic acid ameliorates systemic lupus erythematosus in mice. *The Journal of Clinical Investigation*.

[86] Look M., Saltzman W. M., Craft J., Fahmy T. M. (2014). The nanomaterial-dependent modulation of dendritic cells and its potential influence on therapeutic immunosuppression in lupus. *Biomaterials*.

[87] Zhang Y., Liu S., Yu Y. (2011). Immune complex enhances tolerogenecity of immature dendritic cells via Fc*γ*RIIb and promotes Fc*γ*RIIb-overexpressing dendritic cells to attenuate lupus. *European Journal of Immunology*.

[88] Xia Y., Jiang S., Weng S., Lv X., Cheng H., Fang C. (2011). Antigen-specific immature dendritic cell vaccine ameliorates anti-dsDNA antibody-induced renal damage in a mouse model. *Rheumatology*.

[89] Sela U., Sharabi A., Dayan M., Hershkoviz R., Mozes E. (2009). The role of dendritic cells in the mechanism of action of a peptide that ameliorates lupus in murine models. *Immunology*.

[90] Wu H. J., Lo Y., Luk D., Lau C. S., Lu L., Mok M. Y. (2015). Alternatively activated dendritic cells derived from systemic lupus erythematosus patients have tolerogenic phenotype and function. *Clinical Immunology*.

[91] Santana-de Anda K., Gómez-Martín D., Monsivais-Urenda A. E., Salgado-Bustamante M., González-Amaro R., Alcocer-Varela J. (2014). Interferon regulatory factor 3 as key element of the interferon signature in plasmacytoid dendritic cells from systemic lupus erythematosus patients: novel genetic associations in the Mexican mestizo population. *Clinical and Experimental Immunology*.

[92] Carvalheiro T., Rodrigues A., Lopes A. (2012). Tolerogenic versus inflammatory activity of peripheral blood monocytes and dendritic cells subpopulations in systemic lupus erythematosus. *Clinical and Developmental Immunology*.

[93] Blomberg S., Eloranta M.-L., Magnusson M., Alm G. V., Ronnblom L. (2003). Expression of the markers BDCA-2 and BDCA-4 and production of interferon-*α* by plasmacytoid dendritic cells in systemic lupus erythematosus. *Arthritis & Rheumatism*.

[94] Wu P., Wu J., Liu S. (2008). TLR9/TLR7-triggered downregulation of BDCA2 expression on human plasmacytoid dendritic cells from healthy individuals and lupus patients. *Clinical Immunology*.

[95] Tucci M., Quatraro C., Lombardi L., Pellegrino C., Dammacco F., Silvestris F. (2008). Glomerular accumulation of plasmacytoid dendritic cells in active lupus nephritis: role of interleukin-18. *Arthritis and Rheumatism*.

[96] Vermi W., Lonardi S., Morassi M. (2009). Cutaneous distribution of plasmacytoid dendritic cells in lupus erythematosus. Selective tropism at the site of epithelial apoptotic damage. *Immunobiology*.

[97] Yin Q., Xu X., Lin Y., Lv J., Zhao L., He R. (2014). Ultraviolet B irradiation induces skin accumulation of plasmacytoid dendritic cells: a possible role for chemerin. *Autoimmunity*.

[98] Park J. W., Moon S. Y., Lee J. H. (2014). Bone marrow analysis of immune cells and apoptosis in patients with systemic lupus erythematosus. *Lupus*.

[99] Ito T., Kanzler H., Duramad O., Cao W., Liu Y.-J. (2006). Specialization, kinetics, and repertoire of type 1 interferon responses by human plasmacytoid predendritic cells. *Blood*.

[100] Agrawal H., Jacob N., Carreras E. (2009). Deficiency of type I IFN receptor in lupus-prone New Zealand mixed 2328 mice decreases dendritic cell numbers and activation and protects from disease. *The Journal of Immunology*.

[101] Pau E., Cheung Y.-H., Loh C., Lajoie G., Wither J. E. (2012). TLR tolerance reduces IFN-alpha production despite plasmacytoid dendritic cell expansion and anti-nuclear antibodies in NZB bicongenic mice. *PLoS ONE*.

[102] Zhan Y., Carrington E. M., Ko H. (2015). Bcl-2 antagonists kill plasmacytoid dendritic cells from lupus-prone mice and dampen interferon-alpha production. *Arthritis & Rheumatology*.

[103] Guiducci C., Gong M., Xu Z. (2010). TLR recognition of self nucleic acids hampers glucocorticoid activity in lupus. *Nature*.

[104] Hong Y., Wu J., Zhao J. (2013). miR-29b and miR-29c are involved in Toll-like receptor control of glucocorticoid-induced apoptosis in human plasmacytoid dendritic cells. *PLoS ONE*.

[105] Lepelletier Y., Zollinger R., Ghirelli C. (2010). Toll-like receptor control of glucocorticoid-induced apoptosis in human plasmacytoid predendritic cells (pDCs). *Blood*.

[106] Bonaccorsi I., Cantoni C., Carrega P. (2010). The immune inhibitory receptor LAIR-1 is highly expressed by plasmacytoid dendritic cells and acts complementary with NKp44 to control IFN*α* production. *PLoS ONE*.

[107] Kanakoudi-Tsakalidou F., Farmaki E., Tzimouli V. (2014). Simultaneous changes in serum HMGB1 and IFN-*α* levels and in LAIR-1 expression on plasmatoid dendritic cells of patients with juvenile SLE.. New therapeutic options?. *Lupus*.

[108] Garcia-Romo G. S., Caielli S., Vega B. (2011). Netting neutrophils are major inducers of type I IFN production in pediatric systemic lupus erythematosus. *Science Translational Medicine*.

[109] Liao X., Li S., Settlage R. E. (2015). Cutting edge: plasmacytoid dendritic cells in late-stage lupus mice defective in producing IFN-*α*. *The Journal of Immunology*.

[110] Wilson L. E., Widman D., Dikman S. H., Gorevic P. D. (2002). Autoimmune disease complicating antiviral therapy for hepatitis C virus infection. *Seminars in Arthritis and Rheumatism*.

[111] Rönnblom L. E., Alm G. V., Öberg K. E. (1990). Possible induction of systemic lupus erythematosus by interferon-*α* treatment in a patient with a malignant carcinoid tumour. *Journal of Internal Medicine*.

[112] Mathian A., Weinberg A., Gallegos M., Banchereau J., Koutouzov S. (2005). IFN-*α* induces early lethal lupus in preautoimmune (New Zealand Black x New Zealand White) F1 but not in BALB/c mice. *Journal of Immunology*.

[113] Santiago-Raber M.-L., Baccala R., Haraldsson K. M. (2003). Type-I interferon receptor deficiency reduces lupus-like disease in NZB mice. *The Journal of Experimental Medicine*.

[114] Braun D., Geraldes P., Demengeot J. (2003). Type I Interferon controls the onset and severity of autoimmune manifestations in lpr mice. *Journal of Autoimmunity*.

[115] Hron J. D., Peng S. L. (2004). Type I IFN protects against murine lupus. *The Journal of Immunology*.

[116] Baccala R., Gonzalez-Quintial R., Schreiber R. D., Lawson B. R., Kono D. H., Theofilopoulos A. N. (2012). Anti-IFN-*α*/*β* receptor antibody treatment ameliorates disease in lupus-predisposed mice. *Journal of Immunology*.

[117] Sisirak V., Ganguly D., Lewis K. L. (2014). Genetic evidence for the role of plasmacytoid dendritic cells in systemic lupus erythematosus. *The Journal of Experimental Medicine*.

[118] Santer D. M., Wiedeman A. E., Teal T. H., Ghosh P., Elkon K. B. (2012). Plasmacytoid dendritic cells and C1q differentially regulate inflammatory gene induction by lupus immune complexes. *Journal of Immunology*.

[119] Fehr E.-M., Spoerl S., Heyder P. (2013). Apoptotic-cell-derived membrane vesicles induce an alternative maturation of human dendritic cells which is disturbed in SLE. *Journal of Autoimmunity*.

[120] Abeler-Dörner L., Rieger C. C., Berger B. (2013). Interferon-*α* abrogates the suppressive effect of apoptotic cells on dendritic cells in an in vitro model of systemic lupus erythematosus pathogenesis. *The Journal of Rheumatology*.

[121] Yamauchi M., Hashimoto M., Ichiyama K. (2007). Ifi202, an IFN-inducible candidate gene for lupus susceptibility in NZB/ W F1 mice, is a positive regulator for NF-*κ*B activation in dendritic cells. *International Immunology*.

[122] Huang X., Shen N., Bao C., Gu Y., Wu L., Chen S. (2008). Interferon-induced protein IFIT4 is associated with systemic lupus erythematosus and promotes differentiation of monocytes into dendritic cell-like cells. *Arthritis Research & Therapy*.

[123] Hu Y., Ivashkiv L. B. (2006). Costimulation of chemokine receptor signaling by matrix metalloproteinase-9 mediates enhanced migration of IFN-*α* dendritic cells. *The Journal of Immunology*.

[124] Mangini A. J., Lafyatis R., Van Seventer J. M. (2007). Type I interferons inhibition of inflammatory T helper cell responses in systemic lupus erythematosus. *Annals of the New York Academy of Sciences*.

[125] Clement M., Charles N., Escoubet B. (2015). CD4+CXCR3+ T cells and plasmacytoid dendritic cells drive accelerated atherosclerosis associated with systemic lupus erythematosus. *Journal of Autoimmunity*.

[126] Wang J. H., Wu Q., Yang P. (2011). Type I interferon-dependent CD86^high^ marginal zone precursor B cells are potent T cell costimulators in mice. *Arthritis & Rheumatism*.

[127] Li H., Fu Y. X., Wu Q. (2015). Interferon-induced mechanosensing defects impede apoptotic cell clearance in lupus. *The Journal of Clinical Investigation*.

[128] Quan T. E., Roman R. M., Rudenga B. J., Holers V. M., Craft J. E. (2010). Epstein-barr virus promotes interferon-*α* production by plasmacytoid dendritic cells. *Arthritis and Rheumatism*.

[129] Båve U., Magnusson M., Eloranta M.-L., Perers A., Alm G. V., Rönnblom L. (2003). Fc*γ*RIIa is expressed on natural IFN-*α*-producing cells (plasmacytoid dendritic cells) and is required for the IFN-*α* production induced by apoptotic cells combined with lupus IgG. *The Journal of Immunology*.

[130] Means T. K., Latz E., Hayashi F., Murali M. R., Golenbock D. T., Luster A. D. (2005). Human lupus autoantibody-DNA complexes activate DCs through cooperation of CD32 and TLR9. *The Journal of Clinical Investigation*.

[131] Vollmer J., Tluk S., Schmitz C. (2005). Immune stimulation mediated by autoantigen binding sites within small nuclear RNAs involves Toll-like receptors 7 and 8. *The Journal of Experimental Medicine*.

[132] Mold C., Clos T. W. D. (2013). C-reactive protein inhibits plasmacytoid dendritic cell interferon responses to autoantibody immune complexes. *Arthritis and Rheumatism*.

[133] Wang H., Li T., Chen S., Gu Y., Ye S. (2015). Neutrophil extracellular trap mitochondrial DNA and its autoantibody in systemic lupus erythematosus and a proof-of-concept trial of metformin. *Arthritis & Rheumatology*.

[134] Lande R., Gregorio J., Facchinetti V. (2007). Plasmacytoid dendritic cells sense self-DNA coupled with antimicrobial peptide. *Nature*.

[135] Lande R., Ganguly D., Facchinetti V. (2011). Neutrophils activate plasmacytoid dendritic cells by releasing self-DNA-peptide complexes in systemic lupus erythematosus. *Science Translational Medicine*.

[136] Baccala R., Gonzalez-Quintial R., Blasius A. L. (2013). Essential requirement for IRF8 and SLC15A4 implicates plasmacytoid dendritic cells in the pathogenesis of lupus. *Proceedings of the National Academy of Sciences of the United States of America*.

[137] Sadanaga A., Nakashima H., Akahoshi M. (2007). Protection against autoimmune nephritis in MyD88-deficient MRL/lpr mice. *Arthritis and Rheumatism*.

[138] Xu Y., Lee P. Y., Li Y. (2012). Pleiotropic IFN-dependent and -independent effects of IRF5 on the pathogenesis of experimental lupus. *Journal of Immunology*.

[139] Yasuda K., Richez C., Maciaszek J. W. (2007). Murine dendritic cell type I IFN production induced by human IgG-RNA immune complexes Is IFN regulatory factor (IRF)5 and IRF7 dependent and is required for IL-6 production. *The Journal of Immunology*.

[140] Chiang E. Y., Yu X., Grogan J. L. (2011). Immune complex-mediated cell activation from systemic lupus erythematosus and rheumatoid arthritis patients elaborate different requirements for IRAK1/4 kinase activity across human cell types. *The Journal of Immunology*.

[141] Yanai H., Ban T., Wang Z. (2009). HMGB proteins function as universal sentinels for nucleic-acid-mediated innate immune responses. *Nature*.

[142] Tian J., Avalos A. M., Mao S.-Y. (2007). Toll-like receptor 9-dependent activation by DNA-containing immune complexes is mediated by HMGB1 and RAGE. *Nature Immunology*.

[143] Di Domizio J., Dorta-Estremera S., Gagea M. (2012). Nucleic acid-containing amyloid fibrils potently induce type I interferon and stimulate systemic autoimmunity. *Proceedings of the National Academy of Sciences of the United States of America*.

[144] Orellana C., Collado A., Hernandez M. V., Font J., Del Olmo J. A., Munoz-Gomez J. (1995). When does amyloidosis complicate systemic lupus erythematosus?. *Lupus*.

[145] Gaitonde S., Samols D., Kushner I. (2008). C-reactive protein and systemic lupus erythematosus. *Arthritis Care and Research*.

[146] Kirschfink M., Petry F., Khirwadkar K., Wigand R., Kaltwasser J. P., Loos M. (1993). Complete functional C1q deficiency associated with systemic lupus erythematosus (SLE). *Clinical and Experimental Immunology*.

[147] Walport M. J., Davies K. A., Botto M. (1998). C1q and systemic lupus erythematosus. *Immunobiology*.

[148] Lood C., Gullstrand B., Truedsson L. (2009). C1q inhibits immune complex–induced interferon-*α* production in plasmacytoid dendritic cells: a novel link between C1q deficiency and systemic lupus erythematosus pathogenesis. *Arthritis and Rheumatism*.

[149] Son M., Santiago-Schwarz F., Al-Abed Y., Diamond B. (2012). C1q limits dendritic cell differentiation and activation by engaging LAIR-1. *Proceedings of the National Academy of Sciences of the United States of America*.

[150] Berghöfer B., Frommer T., Haley G., Fink L., Bein G., Hackstein H. (2006). TLR7 ligands induce higher IFN-*α* production in females. *Journal of Immunology*.

[151] Li X., Xu Y., Ma L., Sun L., Fu G., Hou Y. (2009). 17beta-estradiol enhances the response of plasmacytoid dendritic cell to CpG. *PloS ONE*.

[152] Berggren O., Hagberg N., Weber G., Alm G. V., Rönnblom L., Eloranta M.-L. (2012). B lymphocytes enhance interferon-*α* production by plasmacytoid dendritic cells. *Arthritis & Rheumatism*.

[153] Duffau P., Seneschal J., Nicco C. (2010). Platelet CD154 potentiates interferon-*α* secretion by plasmacytoid dendritic cells in systemic lupus erythematosus. *Science Translational Medicine*.

[154] Hagberg N., Berggren O., Leonard D. (2011). IFN-*α* production by plasmacytoid dendritic cells stimulated with RNA-containing immune complexes is promoted by NK Cells via MIP-1*β* and LFA-1. *Journal of Immunology*.

[155] Eloranta M.-L., Lovgren T., Finke D. (2009). Regulation of the interferon-*α* production induced by RNA-containing immune complexes in plasmacytoid dendritic cells. *Arthritis & Rheumatism*.

[156] Santer D. M., Hall B. E., George T. C. (2010). C1q deficiency leads to the defective suppression of IFN-*α* in response to nucleoprotein containing immune complexes. *The Journal of Immunology*.

[157] Cederblad B., Blomberg S., Vallin H., Perers A., Alm G. V., Rönnblom L. (1998). Patients with systemic lupus erythematosus have reduced numbers of circulating natural interferon-*α*-producing cells. *Journal of Autoimmunity*.

[158] Sacre K., Criswell L. A., McCune J. M. (2012). Hydroxychloroquine is associated with impaired interferon-alpha and tumor necrosis factor-alpha production by plasmacytoid dendritic cells in systemic lupus erythematosus. *Arthritis Research & Therapy*.

[159] Kwok S.-K., Lee J.-Y., Park S.-H. (2008). Dysfunctional interferon-alpha production by peripheral plasmacytoid dendritic cells upon Toll-like receptor-9 stimulation in patients with systemic lupus erythematosus. *Arthritis Research & Therapy*.

[160] Fabricius D., Neubauer M., Mandel B. (2010). Prostaglandin E_2_ inhibits IFN-*α* secretion and Th1 costimulation by human plasmacytoid dendritic cells via E-prostanoid 2 and E-prostanoid 4 receptor engagement. *The Journal of Immunology*.

[161] Lindau D., Mussard J., Rabsteyn A. (2014). TLR9 independent interferon alpha production by neutrophils on NETosis in response to circulating chromatin, a key lupus autoantigen. *Annals of the Rheumatic Diseases*.

[162] Denny M. F., Yalavarthi S., Zhao W. (2010). A distinct subset of proinflammatory neutrophils isolated from patients with systemic lupus erythematosus induces vascular damage and synthesizes type I IFNs. *The Journal of Immunology*.

[163] Palanichamy A., Bauer J. W., Yalavarthi S. (2014). Neutrophil-mediated IFN activation in the bone marrow alters B cell development in human and murine systemic lupus erythematosus. *Journal of Immunology*.

[164] McDonald G., Cabal N., Vannier A. (2015). Female bias in systemic lupus erythematosus is associated with the differential expression of X-linked Toll-like receptor 8. *Frontiers in Immunology*.

[165] Richez C., Yasuda K., Watkins A. A. (2009). TLR4 ligands induce IFN-*α* production by mouse conventional dendritic cells and human monocytes after IFN-*β* priming. *The Journal of Immunology*.

[166] Lövgren T., Eloranta M.-L., Kastner B., Wahren-Herlenius M., Alm G. V., Rönnblom L. (2006). Induction of interferon-*α* by immune complexes or liposomes containing systemic lupus erythematosus autoantigen- and Sjögren's syndrome auto antigen—associated RNA. *Arthritis and Rheumatism*.

[167] Lee P. Y., Weinstein J. S., Nacionales D. C. (2008). A novel type I IFN-producing cell subset in murine lupus. *The Journal of Immunology*.

[168] Klarquist J., Hennies C. M., Lehn M. A., Reboulet R. A., Feau S., Janssen E. M. (2014). STING-mediated DNA sensing promotes antitumor and autoimmune responses to dying cells. *The Journal of Immunology*.

[169] Wiedeman A. E., Santer D. M., Yan W., Miescher S., Käsermann F., Elkon K. B. (2013). Contrasting mechanisms of interferon-*α* inhibition by intravenous immunoglobulin after induction by immune complexes versus Toll-like receptor agonists. *Arthritis & Rheumatism*.

[170] Lood C., Allhorn M., Lood R. (2012). IgG glycan hydrolysis by endoglycosidase S diminishes the proinflammatory properties of immune complexes from patients with systemic lupus erythematosus: a possible new treatment?. *Arthritis and Rheumatism*.

[171] Lenert P., Yasuda K., Busconi L. (2009). DNA-like class R inhibitory oligonucleotides (INH-ODNs) preferentially block autoantigen-induced B-cell and dendritic cell activation in vitro and autoantibody production in lupus-prone MRL-Faslpr/lpr mice in vivo. *Arthritis Research & Therapy*.

[172] Hirai M., Kadowaki N., Kitawaki T. (2011). Bortezomib suppresses function and survival of plasmacytoid dendritic cells by targeting intracellular trafficking of Toll-like receptors and endoplasmic reticulum homeostasis. *Blood*.

[173] Ichikawa H. T., Conley T., Muchamuel T. (2012). Beneficial effect of novel proteasome inhibitors in murine lupus via dual inhibition of type I interferon and autoantibody-secreting cells. *Arthritis and Rheumatism*.

[174] Amuro H., Ito T., Miyamoto R. (2010). Statins, inhibitors of 3-hydroxy-3-methylglutaryl-coenzyme A reductase, function as inhibitors of cellular and molecular components involved in type I interferon production. *Arthritis and Rheumatism*.

[175] Salvi V., Bosisio D., Mitola S., Andreoli L., Tincani A., Sozzani S. (2010). Trichostatin A blocks type I interferon production by activated plasmacytoid dendritic cells. *Immunobiology*.

[176] Petri M., Wallace D. J., Spindler A. (2013). Sifalimumab, a human anti-interferon-*α* monoclonal antibody, in systemic lupus erythematosus: a phase I randomized, controlled, dose-escalation study. *Arthritis & Rheumatism*.

[177] Kang H.-K., Liu M., Datta S. K. (2007). Low-dose peptide tolerance therapy of lupus generates plasmacytoid dendritic cells that cause expansion of autoantigen-specific regulatory T cells and contraction of inflammatory Th17 cells. *Journal of Immunology*.

[178] Christensen S. R., Shupe J., Nickerson K., Kashgarian M., Flavell R., Shlomchik M. J. (2006). Toll-like receptor 7 and TLR9 dictate autoantibody specificity and have opposing inflammatory and regulatory roles in a murine model of lupus. *Immunity*.

[179] Wu X., Peng S. L. (2006). Toll-like receptor 9 signaling protects against murine lupus. *Arthritis & Rheumatism*.

[180] Santiago-Raber M.-L., Dunand-Sauthier I., Wu T. (2010). Critical role of TLR7 in the acceleration of systemic lupus erythematosus in TLR9-deficient mice. *Journal of Autoimmunity*.

[181] Nickerson K. M., Cullen J. L., Kashgarian M., Shlomchik M. J. (2013). Exacerbated autoimmunity in the absence of TLR9 in MRL.*Fas*
^lpr^ mice depends on *Ifnar1*. *Journal of Immunology*.

